# Evidence for Presence and Functional Effects of Kv1.1 Channels in β-Cells: General Survey and Results from *mceph/mceph* Mice

**DOI:** 10.1371/journal.pone.0018213

**Published:** 2011-04-05

**Authors:** Zuheng Ma, Catharina Lavebratt, Malin Almgren, Neil Portwood, Lars E. Forsberg, Robert Bränström, Erik Berglund, Sture Falkmer, Frank Sundler, Nils Wierup, Anneli Björklund

**Affiliations:** 1 Department of Molecular Medicine and Surgery, Karolinska Institutet, Karolinska University Hospital, Stockholm, Sweden; 2 Laboratory of Pathology and Clinical Cytology, Ryhov Hospital, Jönköping, Sweden; 3 Department of Experimental Medical Science, Lund University, Lund, Sweden; University of Bremen, Germany

## Abstract

**Background:**

Voltage-dependent K^+^ channels (Kv) mediate repolarisation of β-cell action potentials, and thereby abrogate insulin secretion. The role of the Kv1.1 K^+^ channel in this process is however unclear. We tested for presence of Kv1.1 in different species and tested for a functional role of Kv1.1 by assessing pancreatic islet function in BALB/cByJ (wild-type) and *megencephaly* (*mceph/mceph*) mice, the latter having a deletion in the Kv1.1 gene.

**Methodology/Principal Findings:**

Kv1.1 expression was detected in islets from wild-type mice, SD rats and humans, and expression of truncated Kv1.1 was detected in *mceph/mceph* islets. Full-length Kv1.1 protein was present in islets from wild-type mice, but, as expected, not in those from *mceph/mceph* mice. Kv1.1 expression was localized to the β-cell population and also to α- and δ-cells, with evidence of over-expression of truncated Kv1.1 in *mceph/mceph* islets. Blood glucose, insulin content, and islet morphology were normal in *mceph/mceph* mice, but glucose-induced insulin release from batch-incubated islets was (moderately) higher than that from wild-type islets. Reciprocal blocking of Kv1.1 by dendrotoxin-K increased insulin secretion from wild-type but not *mceph/mceph* islets. Glucose-induced action potential duration, as well as firing frequency, was increased in *mceph/mceph* mouse β-cells. This duration effect on action potential in β-cells from *mceph/mceph* mice was mimicked by dendrotoxin-K in β-cells from wild-type mice. Observations concerning the effects of both the *mceph* mutation, and of dendrotoxin-K, on glucose-induced insulin release were confirmed in pancreatic islets from *Kv1.1* null mice.

**Conclusion/Significance:**

Kv1.1 channels are expressed in the β-cells of several species, and these channels can influence glucose-stimulated insulin release.

## Introduction

It is established that voltage-dependent K^+^ channels (Kv) mediate repolarisation of β- cell action potential and thereby abrogate insulin secretion. Elucidation of the molecular mechanisms and the regulation of Kv channels are however complicated by their multitude of forms, each class being recognized to have several subcategories. Alpha subunits form the actual conductance pore and have been grouped into 12 classes named Kv1-12. Among these channels, Kv2.1 has been shown to be robustly functional in β-cells, to the extent that in a number of species, it is regarded as the most influential Kv channel on insulin secretion [1]. A considerable impact of this channel at the level of electrophysiology has also been documented [2].

It is also recognized that Kv2.1 is not the only Kv channel of importance [2]. Kv1.1 has in animal models been found to be functional in brain, gastrointestinal muscle cells, renal tissue and heart [3], [ 4], [ 5]. A functional role for the Kv1.1 channel in β-cells has been questioned and its expression in this tissue has not been consistently detected [6]. However, a systematic search for the Kv.1.1 channels has not been performed, and a role for Kv1.1 in β-cell function and/or development has not, to our knowledge, been tested rigorously.

In this study, we first obtained preliminary results showing that isolated islets of Langerhans from BALB/cByJ-*Kv1.1^mceph/mceph^* mice (called *mceph/mceph*), for which the Kv1.1 protein is truncated and non-functional secreted more insulin than islets from wild type mice to a glucose challenge. We then searched for and found evidence for Kv1.1 expression in islets from several strains of mice as well as in islets from rats and humans. We continued to test for functional and developmental effects of Kv1.1 on β-cells. We found, as expected that a full-length Kv1.1 protein was missing in islets from *mceph/mceph* mice. Upon finding evidence for mutation-linked effects on insulin secretion we performed electrophysiological measurements to test for traits associated with Kv channel deficiency. Finally, analyses of mice in which the *Kv1.1* gene was knocked out (BALB/cByJ.C3HeB/FeJ-*Kv1.1^−/−^*) confirmed some of the key results in this study.

## Materials and Methods

### Ethics statement

All animal studies were approved by the Northern Stockholm Ethical Committee on Experimental Animal Care (376/03; 348/06) and performed in accordance with guidelines from the Swedish National Board for Laboratory Animals. Human islets were obtained from a Nordic network under conditions specified elsewhere [7].

### Reverse-transcription polymerase chain reaction (PCR)

Total RNA was isolated using RNeasy kits, reverse transcribed and analyzed by conventional and real-time PCR as described in Supporting Information.

### Microarray

Human islets were obtained from three normal individuals and cultured for 48 h in 5.5 mmol/l glucose before RNA was isolated. Labelled cRNAs were synthesized and hybridised to the Human Gene 1.0 ST Array (Affymetrix, Santa Clara, CA) at the Novum Affymetrix core facility, Karolinska Institutet.

### *mceph/mceph* and Kv1.1 null mice

The BALB/cByJ-*Kv1.1^mceph/mceph^* mouse (called *mceph/mceph*, [8] displays temporal lobe epilepsy from a deletion in *Kv1.1* resulting in the expression of a truncated Kv1.1 protein (MCEPH) retaining only the cytosolic N-terminal region, the first transmembrane segment and the first extracellular loop, whilst the voltage sensor and the ion pore domains are lost [9], [10], [11]. In the normal brain, Kv1.1 forms tetramers with other Kv1 subunits via the N-terminal and first transmembrane domain [12], creating channels that regulate neuronal excitability and signalling [13]. *Kv1.1* null mice on BALB/cByJ background show an epileptic behaviour similar to *mceph/mceph* mice [14].

The inbred strains BALB/cByJ-*Kv1.1^mceph/mceph^* BALB/cByJ-*Kv1.1* ^+/+^ and C3HeB/FeJ-*Kcna1^tm1Tem^* were originally obtained from The Jackson Laboratory, Bar Harbor, ME). Fully congenic BALB/cByJ.C3HeB/FeJ-*Kv1.1^−/−^* (*Kv1.1* null) mice were generated at Karolinska Institutet. Briefly, C3HeB/FeJ-*Kcna1^tm1Tem^* mice were outcrossed to BALB/cByJ-*Kv1.1* ^+/+^. F1 males/females heterozygous for the *Kv1.1* null locus were backcrossed to the recipient strain BALB/cByJ-*Kv1.1* ^+/+^ for 10 generations; where after heterozygous mating generated homozygous null mice.

### Histopathological and immunohistochemical techniques

Pancreatic glands were dissected away from the surrounding tissues in their entirety. Specimens from the corpus/cauda regions were used for histopathological staining procedures and immunohistochemical (IHC) analyses. For further details see Supporting Information.

### Western blotting

Protein extracts were separated on SDS-PAGE gels, transferred to nitrocellulose filters and probed with monoclonal antibodies as described in Supporting Information.

### Blood collection and isolation and culture of pancreatic islets

Blood collection and isolation of pancreatic islets as well as tissue culture of MIN6 and INS-1 cells, were carried out as described in Supporting Information.

### Insulin release measurements

#### Batch–type incubations

Following culture and preincubation as described in Supporting Information equal sized islets were incubated in groups of three for 60 min at 37°C in 300 µl KRB containing 3.3 or 16.7 mmol/l glucose, each with or without 10 nM dendrotoxin-K. Each experimental condition consisted of three or four individual groups of three islets. The insulin accumulated in the incubation medium was measured as previously described [15]. Islet insulin content was measured after acid-ethanol extraction [16] of islets retrieved from the batch incubations.

#### Perifusion of islets and dispersed islet cells

After culture and preincubation, 60–80 islets or dispersed islet cells from the same amount of islets (see Supporting information) were added to each of two or three perifusion chambers and perifused as previously described [17]. Briefly, islets or cell suspension were layered between polystyrene beads (Bio-Rad) and perifused by use of a peristaltic pump (Ismatec SA, Zürich, Switzerland). Samples of the perifusate were collected every minute, frozen and stored at −20°C.

### Fura-2 imaging

Fura-2 imaging of dispersed islet cells was performed with a camera (CH250/KAF1400, Photometrics, Tucson, AZ, USA) coupled to an inovison imaging system (Durham, NC, USA) using an inverted fluorescence microscope (Axiovert135TV, Zeiss, Göttingen, Germany). For details see Supporting Information.

### Electrophysiology

Whole-cell currents and membrane potentials were recorded with patch-clamp technique [18] by using an EPC-10 patch-clamp amplifier (HEKA Electronics, Lambercht, Germany). All experiments were performed at room temperature, approximately 22°C. For details see Supporting Information.

### Statistical analysis

Results are expressed as mean ± SEM. Significant differences in the means of sample groups from *mceph/mceph* and wild-type mice were tested using Student's paired t- test (two-sided). A *P* value <0.05 was considered significant.

## Results

### Detection of Kv1.1 mRNA expression

**Figure 1:**
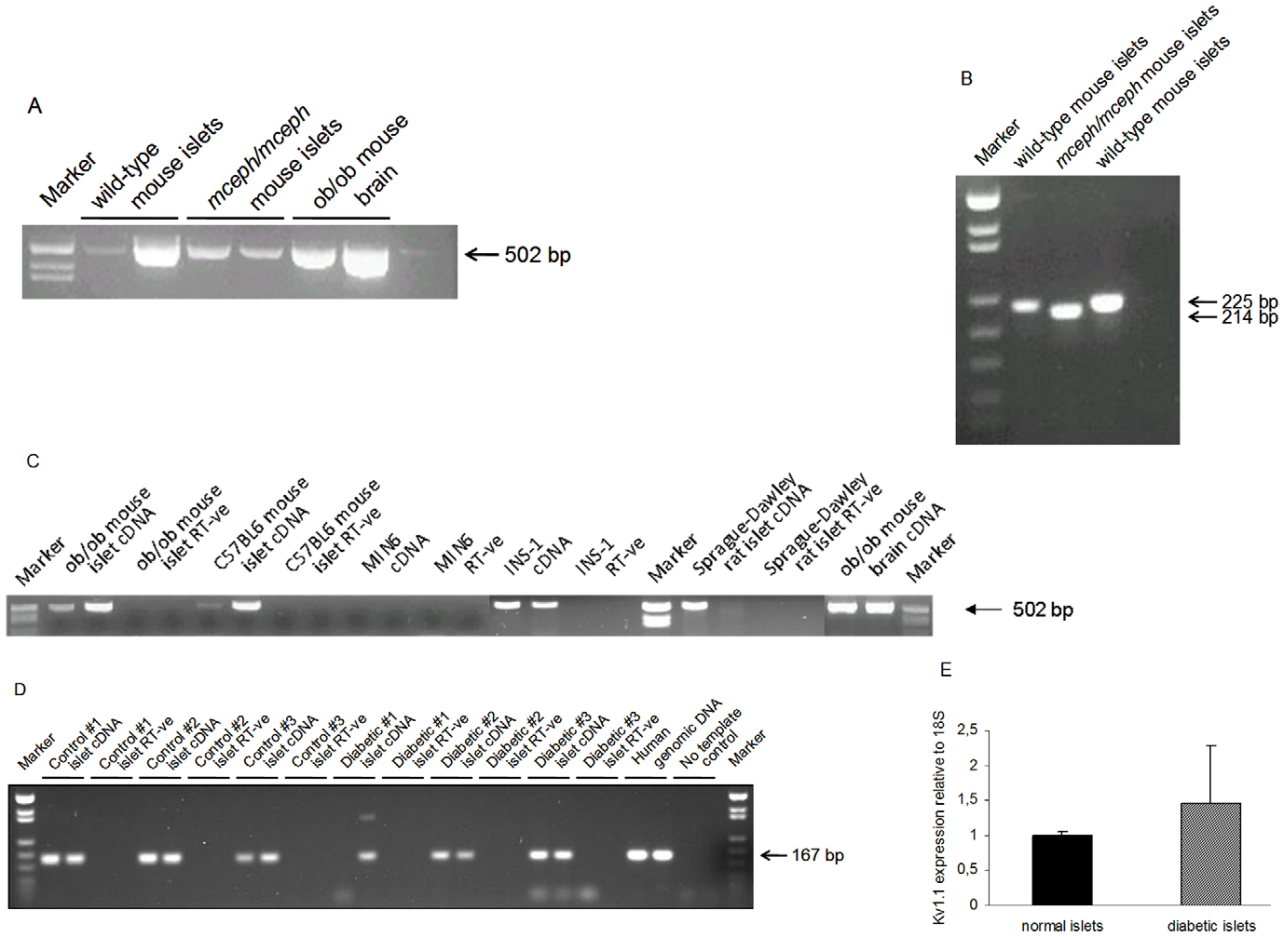
*Kv1.1* is expressed in *mceph/mceph* mice. *Kv1.1* mRNA expression in islets from wild-type and *mceph/mceph* mice by RT-PCR with the S2645-AS3455 (A) and S2688-AS2912 (B) primer pairs, which amplify sequences not including, and including, the *mceph* mutation respectively. Marker = pUC18 Msp I digest. ***Kv1.1*** **is expressed in mice, rat and human islets as well as INS-1 cells but not MIN6 cells**. *Kv1.1* mRNA expression by RT-PCR with the S2645-AS3455 primer pair (C). Marker = pUC18 Msp I digest. (D) *Kv1.1* mRNA expression in islets isolated from three human control and three diabetic individuals by RT-PCR with the S1926-AS2092 primer pair. Marker = pUC18 MspI digest. (E) *Kv1.1* mRNA expression levels relative to 18S RNA by real-time PCR in islets from human non-diabetic (n = 3) and diabetic individuals (n = 3). Samples were run in duplicates. All analyses included a corresponding negative control (RT-ve).

We demonstrated Kv1.1-specific expression using cDNA from wild-type and BALB/cByJ-*Kv1.1^mceph/mceph^* mice (*mceph/mceph*), for which the Kv1.1 protein is truncated and non-functional (Fig. 1A). One of the amplicons employed covered the *mceph* mutation and accordingly differed in size between *mceph/mceph* and wild-type islets (Fig. 1B).

We detected Kv1.1 expression both in islets from *ob/ob* and lean mice, as well as in islets from rat (Fig. 1C). Kv1.1 expression was also detected in the INS-1 cell line, but not in MIN6 cells (Fig. 1C). Importantly we in addition detected Kv1.1 expression in human islets isolated from both non-diabetic donors and donors with type 2 diabetes (Fig. 1D). Using real-time PCR no differences in Kv1.1 expression levels (relative to 18S) were observed between islets from three non-diabetic and three diabetic individuals. However, the results demonstrated a greater variability in Kv1.1 expression in islets from donors with type 2 diabetes (1.0±0.05 vs. 1.5±0.8, *P* = 0.7, n = 3, Fig. 1E).

Kv1.1 expression was also verified in human islets from three non-diabetic individuals by microarray (Affymetrix, results not shown).

Hence, *Kv1.1* expression was detectable in normal islet tissue from rats, humans and mice.

### Detection of Kv1.1 protein

**Figure 2:**
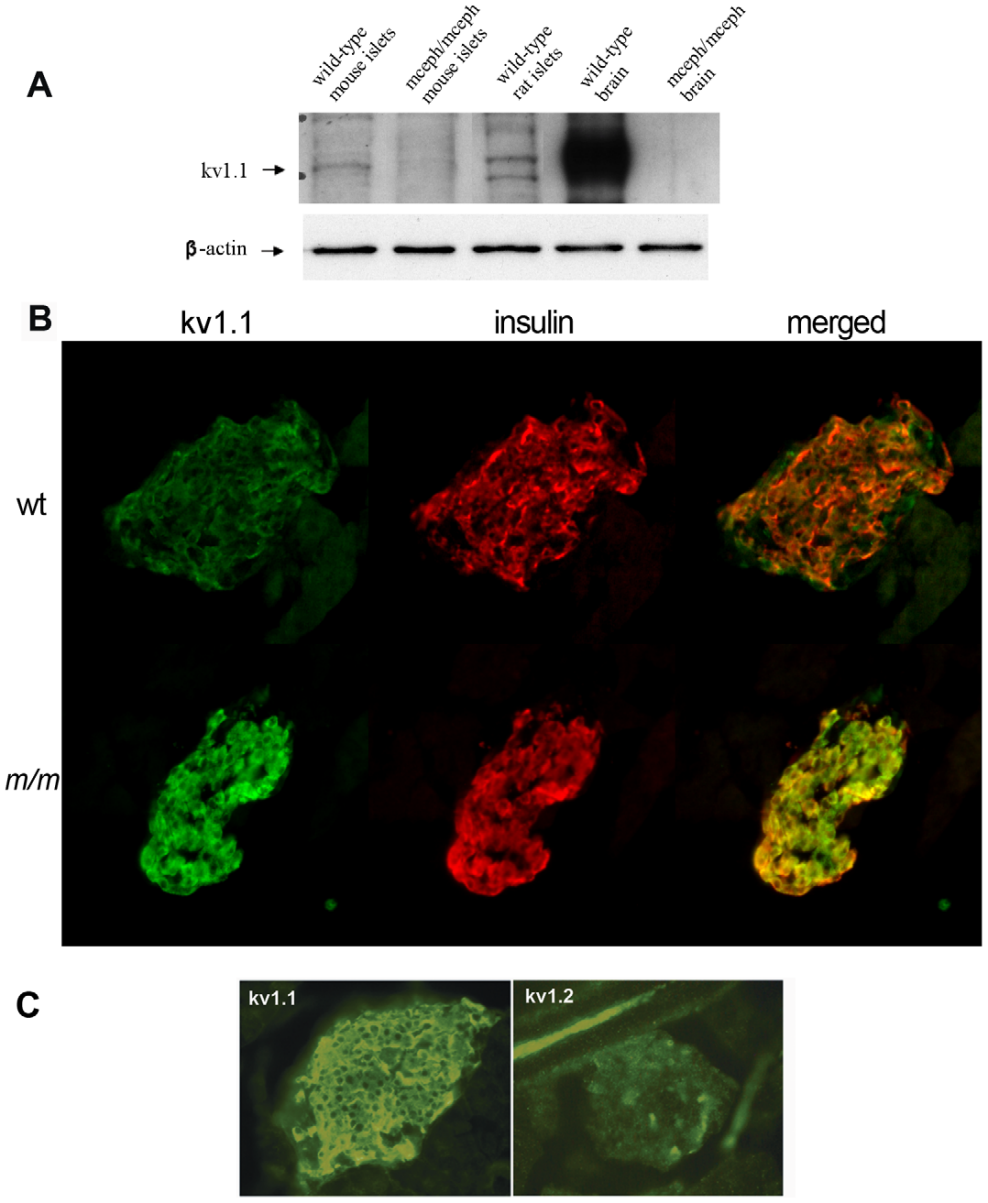
Kv1.1 protein is present in islets from wild type mice and rats. Western blotting using a monoclonal antibody against the C-terminal of Kv1.1 protein (A). Brain from wild type and *mceph/mceph* mice was used as controls. *mceph/mceph* served as specificity control as these mice express only the N-terminal of Kv1.1. β-actin was used as loading control. **Kv1.1 protein is present in β-cells**. Islets from wild type mice (wt) (B upper panel) and *mceph/mceph* (*m/m*) mice (B lower panel) double immunostained for N-terminal Kv1.1 (green, left) and insulin (red, middle); merged to the right. The N-terminal of Kv1.1 is present in beta cells of both genotypes. Note that Kv1.1 is up-regulated in *mceph/mceph* mice compared to wild type mice. Specificity of the N-terminal Kv1.1-LI was tested by determining the level of Kv1.2-LI. Strong N-terminal Kv1.1-LI and very weak Kv1.2-LI in an islet from *mceph/mceph* mice assured that the Kv1.1-LI reflected presence of Kv1.1 (C).

By Western blotting using a monoclonal antibody against the C-terminal of Kv1.1 protein, the full-size Kv1.1 protein was demonstrated to be present in islets from wild-type mice and rats (Fig. 2A), but, as expected, could not be detected in islets from *mceph/mceph* mice which express only the N-terminal of Kv1.1 protein (Fig. 2A).

To investigate in which islet cell types the Kv1.1 protein was present we performed double immunostaining for Kv1.1 and insulin, as well as for glucagon and somatostatin. The Kv1.1 antibody used was against the N-terminal region present in both the normal and the truncated proteins. A clear-cut Kv1.1-like immunoreactivity was found to be co-localized with insulin in the β- cells both in wild-type and in *mceph/mceph* mice. The immunoreactivity was moderate in islets of wild-type mice (Fig. 2B, upper panel, green), but strong in islets of mutated *mceph/mceph* mice (Fig. 2B, lower panel). Hence, the IHC data suggest over-expression of the truncated protein in *mceph/mceph* islets.

We also found co-localization of Kv1.1 with glucagon in the α-cells as well as with somatostatin in δ-cells (data not shown).

The peptide, used to generate the N-terminal Kv1.1 antibody, shares an identity with Kv1.2 consisting of a stretch of 6 out of 24 amino acids. No signal was elicited when islets from *mceph/mceph* mice were incubated with an antibody against Kv1.2. This observation indicates that the signal elicited by the Kv1.1 antibody was due to the truncated (over-expressed) Kv1.1 protein and not due to cross-reactivity with Kv1.2 (Fig. 2C).

### Islet structure in *mceph/mceph* mice

**Figure 3:**
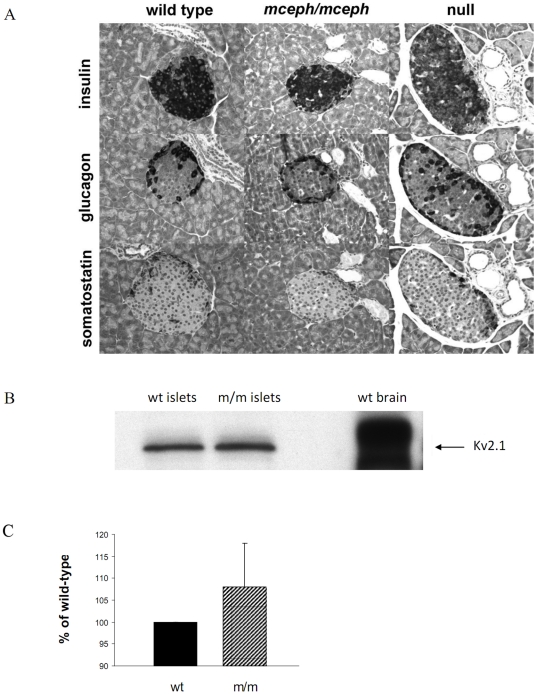
Normal islet structure in *mceph/mceph* and *Kv 1.1* null mice compared to wild type mice. Medium-power photomicrographs, showing- all at the same magnification x400-serial sections of an islet of Langerhans in the pancreatic parenchyma from mice belonging to the three strains (A). The fact that the islet chosen for this particular set of photomicrographs happens to be larger from the Kv1.1 null mouse strain than those from the other two strains, is by pure incidence only, and does not, by no means, imply that the Kv1.1 null islets in general would be larger than those in a normal murine pancreas. **Over-expression of truncated Kv1.1 protein in** ***mceph/mceph*** **does not affect the expression of Kv2.1 protein.** Western blot with a polyclonal antibody against Kv2.1 protein in islets from *mceph/mceph*. Column diagram shows compiled data from 3 individual experiments (C), blots are from a typical experiment (B).

The basic structure of the pancreatic islets of Langerhans was similar for mutant, and wild-type mice, as detected using histopathological staining and IHC analyses. Thus, the typical “mantle” structure of rodent islets was seen, with insulin-immunoreactive cells predominating in the central areas of islets, and glucagon- and somatostatin- imunoreactive cells at the periphery. The proportions of these cell types were 85% insulin-imunoreactive cells, 10% glucagon-imunoreactive cells, and 5% somatostatin- imunoreactive cells (Fig. 3A). There was no evidence of necrosis, inflammation, hypertrophy or atrophy of the three islet parenchymal cell types.

### Detection of Kv 2.1 protein

Over-expression of truncated Kv.1.1 protein in the *mceph/mceph* could affect other Kv channels. To investigate that we performed Western blot for the most important Kv channel involved in repolarisation of action potentials in the beta cell, namely Kv 2.1. No sign of change in the expression of Kv 2.1 channel protein was seen in *mceph/mceph* vs. wild type islets (108±10 vs. 100%, n = 3, Fig. 3B+C).

### Body weights and blood glucose

Body weights were lower in *mceph/mceph* vs. wild-type mice at corresponding ages (Table 1). Levels of blood glucose (non-fasting) did not differ between *mceph/mceph* and wild-type mice (Table 1).

**Table 1:** Age, weight and non-fasting blood glucose values in wild-type, *mceph/mceph* and *Kv1.1* null mice.

	Age (days)	Weight (g)	Glucose (mmol/l)	Animals (n)
Wild type	77±3.5	24±1.0	8.8±0.4	46
*mceph/mceph*	74±2.5	19±0.8*	9.1±0.4	27
*Kv1.1* null	73±4.6	21±1.0*	7.1±0.6* †	18

*P<0.05 vs. wild type.

†*P*<0.05 vs. *mceph/mceph.*

All comparisons were made with One Way ANOVA, Student-Newman-Keuls Method.

### Islet insulin contents and secretion

Insulin contents of isolated islets did not differ between *mceph/mceph* and wild-type islets (1656±194 vs.1509±111 µU/islet, n = 11).

**Figure 4:**
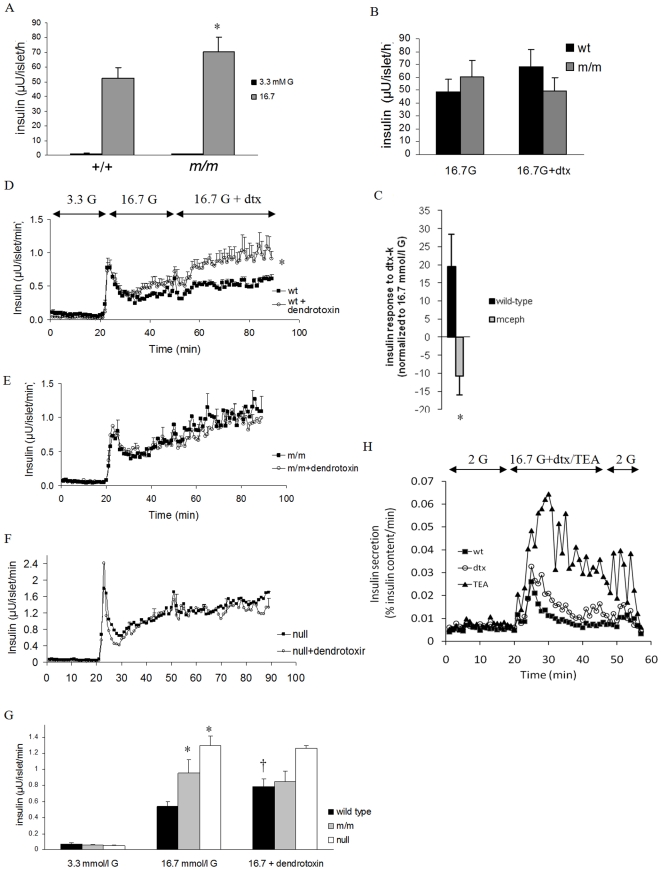
Increased glucose-induced insulin secretion in islets from *mceph/mceph* vs. wild type mice. Glucose-induced insulin secretion from batch-type incubations of islets of Langerhans from wild-type (+/+) and *mceph/mceph* (*m/m*) mice, n = 11 (A). *P<0.03 vs. wild type. **Dendrotoxin-K increases insulin secretion in wild type but not in** ***mceph/mceph*** **and** ***Kv1.1*** **null mouse islets**. Incremental insulin release from batch-type incubations of islets from wild-type (wt) and *mceph/mceph (m/m)* mice in response to 10 nmol/l dendrotoxin-K (dtx), n = 7 (B). The difference in response to dendrotoxin-K for wild-type and *mceph/mceph* mice was significant, *P = 0.02 (C). Each batch-type experiment was performed in triplicate or quadruplicate with three equal-sized islets per tube. Equal amounts of islets from wild-type (D, n = 4), mutant *mceph/mceph* (E, n = 4) or *Kv1.1* null (F, n = 3) mice were loaded into separate perifusion chambers. For typographical reasons only every fifth error bar is shown in (E and F). The response to 10 nmol/l dendrotoxin-K in wild-type islets was significant (*P = 0.05). For details of how the dendrotoxin-K-effect was calculated see results. (G) summarises average insulin response to glucose level elevation from 3.3 to 16.7 mmol/l as well as addition of dendrotoxin-K in the islet perifusion experiments. (The insulin response to an increase in glucose concentration from 3.3 to 16.7 mmol/l was significantly increased in islets from *mceph/mceph* and *Kv1.1* null vs. wild-type mice (*P = 0.01). The response to dendrotoxin-K in wild-type islets was significant (†*P* = 0.05), while that was not the case in *mceph/mceph* and *Kv1.1* null islets. **Increased insulin response to dendrotoxin-K and TEA in dispersed islet cells from wild type mice.** Perifusion with 200 nmol/l dendrotoxin-K or 15 mmol/l TEA. 0.5 mmol/l IBMX was present from min 5 to 51. One typical experiment out of three is shown (H).

Islets from *mceph/mceph* and wild-type mice secreted similar amounts of insulin during culture conditions (596±58 vs. 588±63 µU/islet/24 h). In batch incubations however, glucose (16.7 mmol/l) -induced insulin secretion was moderately although significantly enhanced in islets from *mceph/mceph* mice (71±9.5 vs. 52±7.5 µU/islet/h, respectively, *P*<0.03, n = 11, Fig. 4A). We also performed experiments in the presence of the Kv1.1-specific blocker dendrotoxin-K [19]. Addition of dendrotoxin-K to 16.7 mmol/l glucose-containing media tended to increase insulin secretion from wild type islets (from 49±9.6 to 68±14 µU/islet/h), whereas there was a decrease in release from *mceph/mceph* islets (from 60±13 to 50±10 µU/islet/h, n = 7, Fig. 4B). The difference in response to dendrotoxin-K in wild-type and *mceph/mceph* mice was significant, *P* = 0.02 (Fig 4C).

### Perifusion experiments

Islets from wild type and *mceph*/*mceph* mice were perifused with 3.3 mmol/l glucose followed by 16.7 mmol/l glucose and finally 10 nmol/l dendrotoxin-K was added to the 16.7 mmol/l glucose in one perifusion chamber whereas the other chamber continued to be perifused with 16.7 mmol/l glucose only. The response to dendrotoxin-K was calculated as follows: The average insulin response to 16.7 mmol/l glucose was calculated for min 29–49 and subtracted from the average insulin response at min 69–89 making it possible to calculate the pure effect of dendrotoxin-K in each channel. In wild type islets dendrotoxin-K elicited a significant insulin response of 0.29±0.1 µU/islet/min, *P* = 0.05, Fig. 4D and G) whereas no such response was seen in islets from *mceph/mcpeh* mice (−0.1±0.1 µU/islet/min, *P* = 0.45, Fig. 4E and G). The difference in response between the strains was significant (*P* = 0.035). Also the insulin response to elevating glucose from 3.3 to 16.7 mmol/l during perifusion was significantly increased in islets from *mceph/mceph* vs. wild-type mice (0.34±0.04 vs. 0.51±0.04 µU/islet/min, *P* = 0.01, Fig. 4D, E and G).

We also performed experiments in dispersed islet cells from wild type mice (Fig. 4H). In these experiments basal insulin secretion was similar before stimulation with dendrotoxin-K and TEA (Tetraethylammonium, a non-selective K^+^ channel inhibitor). In the presence of 16.7 mmol/l glucose TEA increased insulin secretion by 148±64%, n = 3, Fig. 4H. A much smaller but still significant effect was obtained by the addition of dendrotoxin-K which increased insulin secretion by 35±9%, *P<* 0.04.

### Fura-2 imaging

We measured [Ca^2+^]_i_ responses to 15 mmol/l glucose ± dendrotoxin-K in dispersed islet cells from *mceph/mceph* (n = 10) and wild type mice (n = 8) using fura-2 imaging. We did not detect any differences between wild type and *mceph/mceph*, (results not shown).

### Electrophysiology

Previous studies in brain tissue found that electrophysiological parameters were affected by the *mceph/mceph* mutation [10]. Furthermore, other Kv deficiencies give rise to excitatory abnormalities in β-cells [2]. Our aim was to see if abnormalities indicative of Kv channel deficiencies and specifically those of Kv1.1, were present in *mceph/mceph* islets.

#### Whole-cell currents

**Figure 5:**
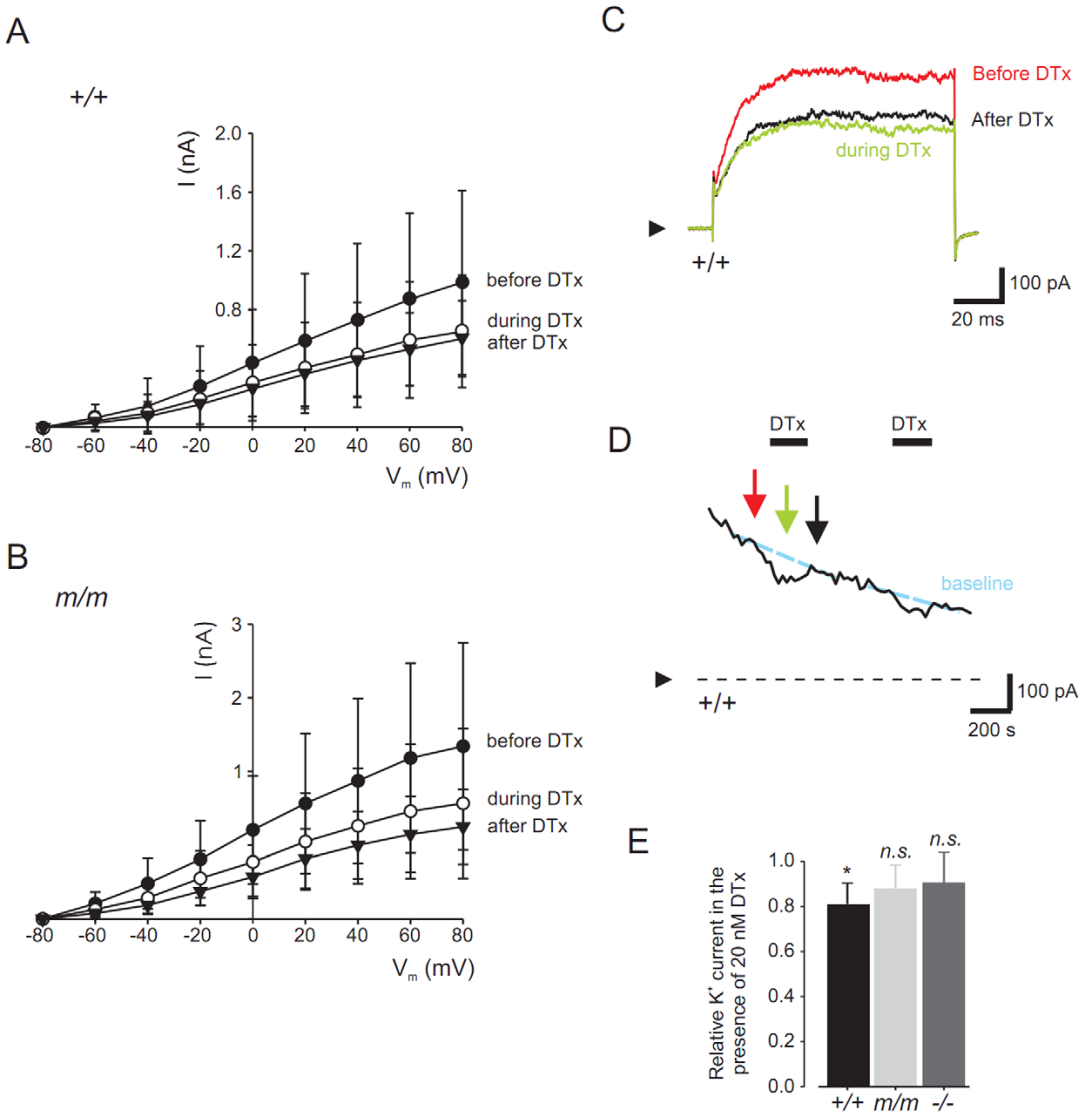
Dendrotoxin-K significantly blocks relative K^+^ currents in wild type vs. *mceph/mceph* β-cells. Whole-cell patch-clamp recordings from wild-type and *mceph/mceph* pancreatic β-cells. *I-V* relationship for wild-type (A) and *mceph/mceph* (B) pancreatic β-cells before, during and after exposure to 20 nmol/l dendrotoxin-K were obtained by clamping the cells at −80 mV and subsequently depolarized in steps of 20 mV for 100 ms to 80 mV. Example traces are recorded from wild-type β-cell in (C) at +80 mV, and the current measured at +80 mV is plotted every 20 s (D). In (E), to compensate for channel run-down, in each recording whole-cell K^+^ current was normalized to the estimated K^+^ current by extrapolating the current before and after the toxin administration.*P<0.05; *n.s.*, not significant.

Whole-cell currents were recorded from pancreatic β-cells isolated from wild-type and *mceph/mceph* mice, using the whole-cell configuration of the patch-clamp technique (Fig. 5A–E). The cells were voltage-clamped at −80 mV and subsequently depolarized in steps of 20 mV every s (Fig. 5A and B). After establishment of the whole-cell configuration, current recordings showed a marked ‘run-down’. To compensate for this ‘run-down’, whole-cell current was measured before and after addition of the Kv1.1 blocker dendrotoxin-K and the effect of the toxin was compared to the estimated whole-cell current. In wild-type β-cells, the K^+^ current was decreased by 20 nmol/l dendrotoxin-K with 20±5% (n = 6; *P*<0.01) and in *mceph/mceph* β-cells with 12±11% (n = 6, *n.s.*). No significant difference could be monitored between the wild-type and *mceph/mceph* β-cell whole cell currents. Compiled data of the effect of dendrotoxin-K on normalized whole-cell K^+^ currents in wild-type and *mecph/mceph* beta cells are shown in Fig. 5E.

#### Membrane potentials

Membrane potentials were monitored using the perforated-patch technique. The membrane potential at 3 mmol/l glucose was −58±4 mV (*n* = 4) in wild-type β-cells, and increased to −41±2 mV (*n* = 5) in the presence of 15 mmol/l glucose (measured as the lowest point between two action potentials). Addition of 20 nmol/l dendrotoxin-K did not affect membrane potential (−39±7 mV, *n* = 3, *n.s.*). In *mceph/mceph* β-cells, membrane potential was −59±5 mV (*n* = 6) and −32±4 mV (*n* = 3) in the presence of 3 mmol/l and 15 mmol/l glucose, respectively. In the presence of 15 mmol/l glucose and 20 nmol/l dendrotoxin-K, the membrane potential was −36±6 mV (*n* = 3, *n.s*). The *mceph/mceph* mice islets were thus more depolarised in 15 vs. 3.3 mmol/l glucose compared to wild type islets (−32±4 vs. −41±2 mV), *P* = 0.018.

#### Action potentials

**Figure 6:**
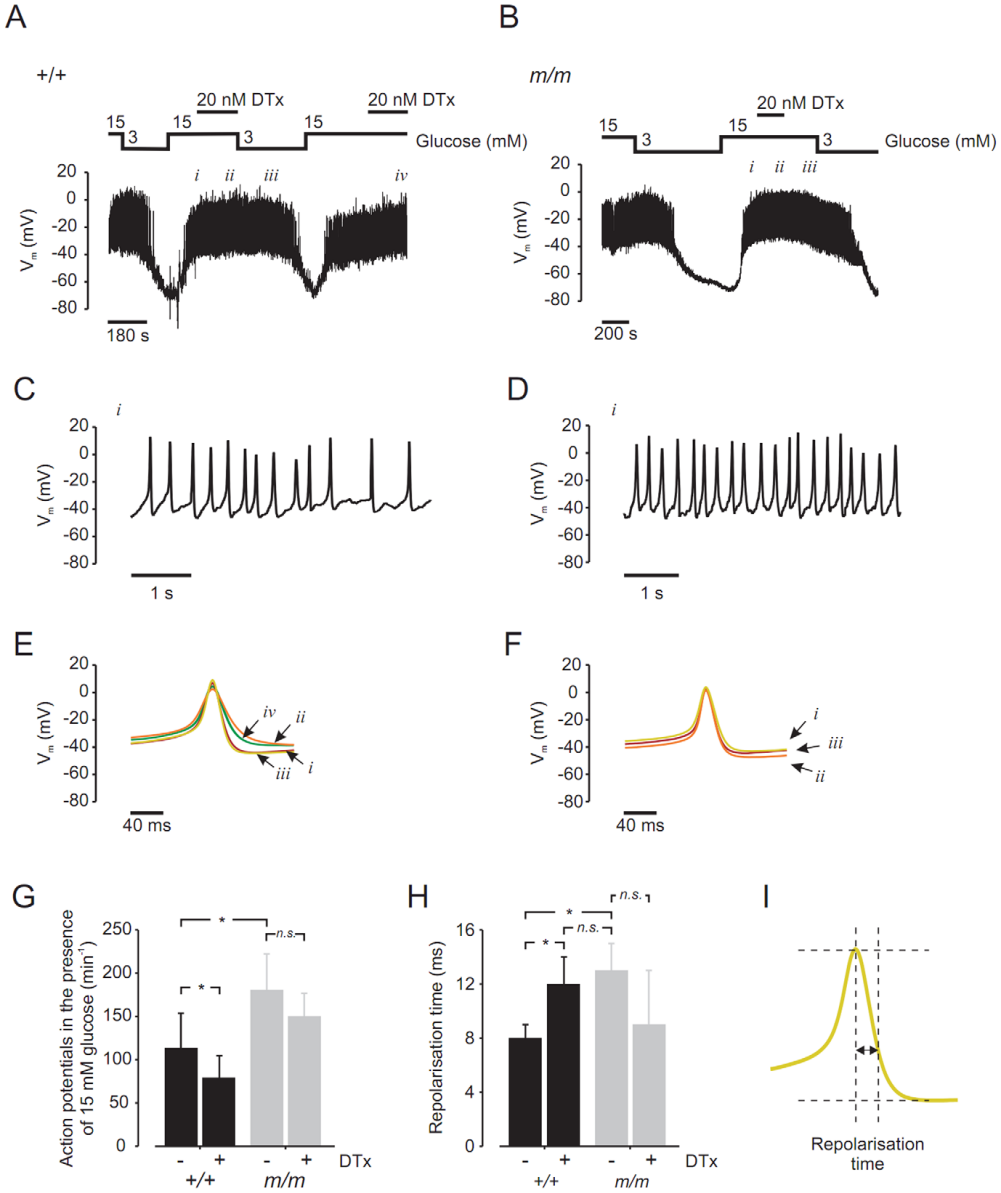
*mceph/mceph* β-cells display a higher action potential frequency and a prolongation of repolarization vs. wild-type β-cells. Membrane potential were recorded in pancreatic β-cells isolated from wild-type mouse (A) and *mceph/mceph* mouse (B). (C) and (D), displays recordings of membrane potential on an expanded time scale during 15 mmol/l glucose (*i*). (E) and (F) show summary of 20 action potentials at 15 mmol/glucose +/−20 nmol/l dendrotoxin-K (indicated by roman numbers *i*, *ii*, *iii* and *iv*). (G), action potential frequency (per 60 s) in 15 mmol/l glucose in β-cells isolated from wild-type (n = 4) and *mceph/mceph* (n = 6), with and without 20 nmol/l dendrotoxin-K (n = 3, both groups). (H) shows compiled data of repolarisation phase in wild-type and *mceph/mceph* β-cells, measured as time lag from action potential peak and 2/3 of action potential amplitude (see schematic drawing in (I)). All analysis of action potential characteristics were performed using sections of membrane potential recording in close proximity and prior to change of solution. *P<0.05; n.s., not significant.

The traces of membrane potential recordings from wild-type (Fig. 6A) and *mceph/mceph* β-cells are shown in Fig. 6B. On an expanded time scale (Fig. 6C–D) details of action potential frequency can be monitored. The frequency of action potentials in wild-type β-cells was significantly lower, compared to that observed in *mceph/mceph* β-cells, whereas the frequency of action potentials decreased in the presence of dendrotoxin-K in wild-type β-cells but remained unaltered in *mceph/mceph* β-cells (Fig. 6G).

#### Detailed analysis of action potential characterizes

Fig. 6E–F show the summary of 20 action potentials obtained in the presence of 15 mmol/l glucose ± dendrotoxin-K. In wild-type β-cells (Fig. 6E), addition of 20 nmol/l dendrotoxin-K prolonged the repolarisation phase (*ii* and *iv*). This effect was reversible upon wash-out of the toxin (*iii*). Augmentation of the repolarization phase was not seen in *mceph/mceph* β-cells (Fig. 6F), in which action potentials remained unaltered in the presence of dendrotoxin-K (Fig. 6F, *i*–*iii*). Fig. 6H shows compiled data of the repolarisation phase in wild-type and *mceph/mceph* β-cells, measured as a time lag from the action potential peak and 2/3 of the action potential amplitude (Fig. 6I). The action potential duration was significantly prolonged in β-cells from *mceph/mceph* mice, compared to those from wild-type mice. Action potential duration was also significantly increased in wild-type β-cells treated with dendrotoxin-K, while dendrotoxin-K did not affect the action potential duration in *mceph/mceph* β-cells.

Collectively, the electrophysiological recordings show that dendrotoxin-K is able to block a fraction of the total K^+^ current in wild-type β-cells, whereas this effect is not present in β-cells from the *mceph/mceph* mouse. Detailed analyses of these action potentials showed that *mceph/mceph* β-cells display a higher action potential frequency and a prolongation of repolarization, compared to wild-type β-cells. This effect on action potential duration could be mimicked by dendrotoxin-K in wild-type β-cells.

### Results in Kv1.1 null mice

With limited access to *Kv1.1* null mice we tested for confirmation of some of the findings in *mceph/mceph*. There were no differences in islet structure in *Kv1.1* null mice, compared to wild-type and *mceph/mceph* (Fig. 3A). In perifusion experiments with islets from wild-type and *Kv1.1* null mice, in which the glucose concentration was increased from 3.3 to 7 mmol/l, insulin secretion was significantly increased in *Kv1.1* null islets vs. wild-type islets (0.47±0.06 vs. 0.19±0.05 µU/islets/min, n = 3 and 4 experiments, *P* = 0.037). The average insulin response from islets when glucose levels rose from 3.3 to 16.7 mmol/l in perifusion experiments was significantly elevated in both *mceph/mceph* and in *Kv1.1* null islets vs. wild type islets (Fig. 4E, F and G).

Also, the lack of effect of dendrotoxin-K in *mceph/mcpeh* islets was confirmed in *Kv1.1* null islets (Fig. 4F and G). Blood glucose levels were significantly lower in *Kv1.1* null animals compared to wild type and *mceph/mceph* (Table 1). Plasma insulin levels displayed a tendency towards higher insulin levels in the *Kv1.1* null mice (3.48±2.3 ng/ml, n = 6) vs. wild type (1.83±0.7 ng/ml, n = 8) and *mceph/mceph* (1.18±0.9 ng/ml. n = 6) mice. Patch clamp recordings showed that in *Kv1.1* null β-cells, the K^+^ current was non-significantly decreased by 20 nmol/l dendrotoxin-K with 10±15% (n = 6; *n.s,* Fig. 5E).

## Discussion

We demonstrate the presence of Kv1.1-specific transcripts in mouse, rat and human islets. Previous reports are discrepant as to the expression of Kv1.1 in primary pancreatic β cells [1], [20], [21]. Discrepancies may be due to the sensitivity of the techniques used. Here we examined Kv1.1 expression by an optimized PCR methodology with appropriate negative controls in all cases. Consistent Kv1.1 amplification from cDNA prepared from rodent islets required 40 cycles of amplification from template levels corresponding to 50 ng total RNA. This indicates that this gene is expressed, albeit at low levels, in mouse and rat islets. Notably, in human islets prepared from six individuals (three controls, three diabetics), Kv1.1 expression was detectable in each sample at template levels corresponding to 10 ng total RNA, and these observations were confirmed by TaqMan real-time PCR and cDNA microarray (data not shown). A previous study did not detect Kv1.1 expression in human islets, however, in that study islets from only a single individual were analyzed [22].

We found that pancreatic islets from *mceph/mceph* mice secrete more insulin in response to glucose compared to islets from wild type mice. The confirmation of these findings in *Kv1.1* null mice indicates that the increase in insulin secretion is indeed due to the lack of functional Kv1.1 channels. The effects of the Kv1.1 blocker dendrotoxin-K on insulin release also strongly indicate that an enhanced insulin response to glucose in the *mceph/mceph* mice is due to a lack of functional Kv1.1 activity. Thus, both in static incubations (islets) and in perifusion experiments (islets and dispersed islet cells), dendrotoxin-K augmented insulin secretion from wild-type mice but not from *mceph/mceph* mice. Perifusion experiments with islets from *Kv1.1* null animals further confirmed these findings.

Our experiments give further indications of the specific impact of the Kv1.1 channel mutation on membrane electrophysiology of β-cells from *mceph/mceph* mice. Importantly, glucose-induced action potentials were increased in spiking frequency and duration in *mceph/mceph* mice when measured in the absence of dendrotoxin-K. The fact that *mceph/mceph* mice β-cells showed an increased action potential frequency at the same time as the action potential duration was increased may seem hard to understand since one would expect a slower frequency (2). At 15 mmol/l glucose there was an increase in membrane potential in *mceph/mceph* vs. wild type β-cells (−32±4 vs. −41±2 mV, *P* = 0.018) which could at least in part explain this finding. Increased action potential duration has also been observed in ventricular myocytes from the hearts of Kv1DN mice with over-expression of a truncated Kv1.1 23. Our results with dendrotoxin-K were in further agreement with the Kv1.1 channel being functional at the level of electrophysiology. Thus, in wild type β-cells dendrotoxin-K obliterated the differences between wild-type and the *mceph/mceph* mice regarding β-cell action potential duration.

Our results concerning expression of the Kv1.1 mRNA and protein support a key role for the Kv1.1 mutation in the abnormal function of β-cells from *mceph/mceph* mice. Kv1.1 expression was thus readily detected in wild-type islets, whereas a truncated form was found in *mceph/mceph* islets. Interestingly, the Kv1.1 protein seemed, in its truncated form in *mceph/mceph* islets, more abundant than the normal protein in islets of wild-type mice. These observations are similar to those reported for some regions in the brain of *mceph/mceph* mice. Accumulation of truncated protein could theoretically have untoward and toxic effects. However, we note that there were, by histological analysis, no signs of β-cell toxicity in *mceph/mceph* mice. Furthermore, there was no sign of change in the expression of Kv2.1 protein. Ablation of Kv2.1 also affected action potentials in a way that was not observed here [2].

Immunostaining showed that Kv1.1 was present also in α- and δ-cells. The functional role, if any, for Kv1.1 in these cells remains to be investigated.

The significantly lower levels of blood glucose and the tendency for higher serum insulin levels in *Kv1.1* null mice vs. wild type seem logical. In contrast *mceph/mceph* mice exhibit blood glucose and insulin levels that are similar to those seen in wild-type mice. *mceph/mceph* and *Kv1.1* null mice have a similar behavioural phenotype with body tremor, jittering and occasional forelimb paddling, and cramps. However, the phenotype of *mceph/mceph* mice appears more severe than that of the *Kv1.1* null mice (Lavebratt, unpublished observations). More marked severity of *Kv1.1* deficiency phenotype could lead to a higher stress level which in turn could result in higher blood glucose levels and increased insulin resistance. In vivo stress could also activate alpha-adrenergic receptors on the β-cell and thereby inhibit insulin secretion thus counteracting the increased insulin secretion from islets when tested in vitro.

Another possibility is that lack of Kv1.1 in the CNS induces problems of glucose sensing in the hypothalamus that could secondarily affect the endocrine pancreas in different ways in the *mceph/mceph* and Kv1.1 null mice. The truncated Kv1.1 in *mceph/mceph* mice can assemble with other Kv1 subunits and potentially trap them in endoplasmatic reticulum with subsequent degradation [11].

Which importance should then be assigned to the Kv1.1 channel for β-cell function vis-à-vis other Kv channels? A large body of evidence indicates that the Kv2.1 channel is the major K^+^-rectifying channel in β-cells. Nevertheless, as highlighted by a recent study [2] Kv2.1 is not the only Kv channel of importance. Hence a supplementary role for Kv1.1 channel (as well as perhaps other Kv channels) can be envisaged. Notably, our results indicate that this notion can be extended also to human beta cells.

In summary Kv1.1 channels display an inter-species expression pattern. Furthermore, our results in mouse β-cells indicate that these channels are of functional importance.

## Supporting Information

Supporting Information S1 (DOC)
