# Supplementary material for: Evidence for Presence and Functional Effects of Kv1.1 Channels in β-Cells: General Survey and Results from mceph/mceph Mice
Source: PLoS One. 2011 Apr 5;6(4):e18213. doi: 10.1371/journal.pone.0018213 (PMC3071710; doi:10.1371/journal.pone.0018213)
Supplement: Supporting Information S1 — (DOC) [file pone.0018213.s001.doc]

**Supporting Information**

Ma et al: Evidence for Presence and Functional Effects of Kv1.1 channels in β-cells: General Survey and Results from *mceph/mceph* mice.

**Materials and Methods**

*Reverse-transcription polymerase chain reaction (PCR)*

Total RNA was isolated from mouse, rat and human islets using RNeasy Micro and Mini kits (Qiagen; Hilden, Germany), respectively. Total islet RNA was treated with DNase I (Promega; SDS Biosciences, Falkenberg, Sweden, or Qiagen), repurified with Mini kits, and reverse-transcribed with SuperScript II (Invitrogen; Lidingö, Sweden), according to the manufacturer’s instructions; reverse transcription (RT) reactions contained 250 (mouse islets) or 50 (human islets) ng/μl total RNA, 5 ng/μl random hexamer primers (GE Healthcare; Uppsala, Sweden), and 2U/μl RNaseOut (Invitrogen). Parallel reactions from which SuperScript II was omitted were employed as RT-negative controls. PCR reactions (volume 10 μl) were set up in duplicate for each template and contained 200 μmol/L each dNTP (GE Healthcare), 5 pmol/L of primers, 0.4U *Taq* polymerase (GE Healthcare), and quantities of cDNA equivalent to 10 ng (human islet, brain, intestine) or 50 ng (rodent islet) RNA. The sequences of primers targeting a region of mouse *Kv1.1 (Kcna1*) mRNA ([NM_010595](http://www.ncbi.nlm.nih.gov/entrez/viewer.fcgi?db=nucleotide&val=31560569)) not including the *mceph* mutation were as follows: S2954, 5’-GTC ATC CGC TTG GTA AGG-3’; AS3455 5’-CCT CTT CGA TCT CCA TGT ACT C-3’; cycling conditions were 94°C 3 mins; 94°C 30 secs, 62°C 30 secs, 72°C 60 secs (35 cycles); 72°C 5 mins. The sequences of primers targeting a region including the *mceph* mutation were as follows: S2688, 5’-GCA TCG ACA ACA CCA CAG TC-3’; AS2912 5’-TTC CCT CCT GCT CAG CTA TC-3’; cycling conditions were 94°C 3 mins; 94°C 30 secs, 58.1°C 30 secs, 72°C 30 secs (40 cycles); 72°C 5 mins. The sequences of primers targeting human Kv1.1 mRNA were as follows: S1926, 5’-CTG AGC AGG AAG GAA ACC AG-3’; AS2092, 5’-GAG CAG CCC TAG CTC TCT CA-3’; cycling conditions were 94°C 3 mins; 94°C 30 secs, 60.8°C 30 secs, 72°C 30 secs (40 cycles); 72°C 5 mins. Human genomic DNA (0.2 ng/PCR reaction) was employed as a positive control template for these PCRs. PCR products were analyzed by agarose gel electrophoresis and documented by digital camera (EDAS 290, Kodak). All PCRs included RT-negative controls, and no products were obtained from these reactions.

For real-time PCR, DNase–treated islet total RNA was reverse-transcribed using TaqMan® Reverse Transcription Reagents (Applied Biosystems; Stockholm, Sweden) according to the manufacturer’s instructions. Real-time PCR reactions contained 1x Universal Master Mix (Applied Biosystems) and Kv1.1 TaqMan® Gene Expression Assay (Applied Biosystems), and were performed using a 7300 Real Time PCR System (Applied Biosystems). Normalization of Kv1.1 levels was carried out by amplification of 18S (Applied Biosystems) from the same templates.

*Histopathological and immunohistochemical techniques.*

Pancreatic glands were dissected away from the surrounding tissues in their entirety. Specimens from the corpus/cauda regions were formalin-fixed, conventionally dehydrated and subsequently paraffin-embedded. Four-micrometer-thick sections were used for histopathological staining procedures and immunohistochemical (IHC) analyses.

Histopathological stainings included haematoxylin-eosin, van-Gieson, and staining for the parenchymal cells of the islets of Langerhans with aldehyde-fuchsin to stain insulin-producing β-cells, and Grimelius silver nitrate to detect non-β-cells, mainly α- cells.

The IHC analyses on paraffin-embedded sections were performed by means of the conventional Daco Envision technique, applying commercially available murine antisera raised against the three major peptide hormones produced in the corpus/cauda part of the pancreas, *viz.* insulin, glucagon, and somatostatin. In addition, antisera against markers for neuroendocrine cells, *viz.* chromogranin A and synaptofysin, were applied (Fig.3A).

A strict, comprehensive, morphometric analysis of the the islet parenchyma in the pancreatic glands of the three mouse genotypes was not accomplished. Already at an early stage of the histopathological examination it became clear that any major, fundamental differences did not seem to exist between any of all the animals investigated. Their pancreatic islets all conformed to those of completely normal mice with regard to localization and distribution in the acinar pancreatic parenchyma, number, size, structure, and cellular composition. This was evaluated by means of direct measurements with an ocular micrometer and simple cell counting in a light microscope after histological and immunostainings. The number of islets analyzed averaged 20 in each cut section. The medians and ranges in islet diameter and in relative incidence of the three major cell types were calculated for each of three mouse genotypes. Particular attention was paid to the occurrence of hypertrophy or atrophy, necrotic areas, and infiltration of inflammatory cells.

Additional mice (Fig. 2B and C) were anesthetized using isofluran and perfused via the ascending aorta with 4% paraformaldehyde. Pancreata were rapidly dissected, immersed in fixative and snap-frozen using dry ice. Sections of 14 µm thickness were cut on a cryostat microtome and thaw-mounted on chromalum gelatin-coated glass slides. IHC was performed as previously described [1]. The primary antibodies used were rabbit anti-Kv1.2 IgG (1:300, Sigma-Aldrich), rabbit anti- N-terminal Kv1.1 IgG (1:300, [1]) and guinea pig anti-proinsulin (code 9003; dilution 1:2560; EuroDiagnostica, Malmö, Sweden). Secondary antibodies were anti-rabbit IgG and anti-guinea pig IgG coupled to either fluorescein isothiocyanate (FITC) or Texas-Red (Jackson, West Grove, PA). Results were obtained from duplicate or triplicate experiments.

*Western blotting*

Equal numbers of islets and brain extract for each experimental condition were collected and washed twice with ice-cold PBS. Extracts corresponding to 100 islets (about 20 μg protein as confirmed by Bradford protein assay) were denatured in 50 μl loading buffer at 80°C for 10 min. Samples were analyzed on 10% SDS–PAGE gels run for 1 h at 150 V and were then transferred to nitrocellulose for 1 h at 250 mA. Membrane were blocked for 2 h at room temperature with 5% (w/v) fat-free milk, 0.1% Tween 20 in Tris-buffered saline, pH 7.6 and then incubated overnight at 4°C with monoclonal mouse anti-C-terminal Kv1.1 IgG (1:1000, Upstate Biotechnologies, Waltham, MA, USA), monoclonal mouse anti-beta-actin IgG (1:5000, Sigma-Aldrich) and polyclonal rabbit anti-Kv2.1 (1:500, Alamone). Second antibody incubations employed an HRP-linked anti-mouse antibody or anti-rabbit for 1 h at room temperature. Immunoreactive bands were visualized using chemiluminescence (ECL Western blotting reagent, Pierce, Biotechnology, USA.

*Blood collection from mceph/mceph mice.*

*mceph/mceph* and wild-type mice were euthanized and weighed and blood was collected for glucose and insulin determinations by cardiac puncture. Blood glucose measured using a device from ACCU-CHEK (Roche, Germany). Serum insulin was measured with an ultrasensitive rat/mouse insulin ELISA kit from Crystal Chem Inc. (Downers Grove, Ilinois, USA).

*Tissue culture.*

The β cell line MIN6 was maintained in DMEM (11 mM glucose) supplemented with 15% fetal calf serum, 100 U/ml penicillin, 0.1 mg/ml streptomycin sulfate, 50 μM β-mercaptoethanol, in a humidified atmosphere of 5% CO_2_ at 37 C.

INS-1 cells were cultured in RPMI 1640 (Invitrogen) supplemented with 10 % fetal calf serum and other additions as described previously [2].

*Isolation, incubation and perifusion of pancreatic islets and dispersed islet cells.*

Islets of Langerhans were isolated from euthanized wild-type, mceph/mceph and ob/ob mice, and from Sprague-Dawley (SD) - rats, by collagenase digestion in HBSS as described previously [3], followed by sedimentation. Islets were then selected under stereomicroscope and transferred to Petri dishes (Sterilin, Teddington, UK) containing RPMI-1640, 2 mmol/l glutamine, 10 % (v/v) fetal calf serum, 100 U/ml benzylpenicillin, 0.1 mg/ml streptomycin and 11 mmol/l glucose. Islets were cultured free-floating for 20–24 h at 37°C, in an atmosphere of 5% CO_2_ in air. Thereafter, equal-sized islets were transferred to dishes containing 5 ml Krebs–Ringer bicarbonate (KRB) medium, 10 mmol/l Hepes, 0.2% BSA, and 3.3 mmol/l glucose, and preincubated for 30 min at 37°C. They were then collected for batch incubation, perifusion, RNA extraction or Western blot as described below. A cell suspension was prepared from islets by trypsin digestion as described previously [4] and used in perifusion and patch clamp experiments. The cells were suspended in RPMI 1640 culture medium (see above). For Fura-2 imaging and patch clamp experiments the cell suspension was seeded onto coverslips, and the cells allowed to attach for 2 h and then cultured for up to 2 days in the above medium.

*Fura-2 imaging*

Dispersed islet cells on glass coverslips were loaded with fura-2 AM (3 µmol/l for 30 min in 37ºC). Loaded cells were transferred to an open perifusion chamber where the coverslips formed the bottom. A peristaltic pump controlled the perifusion rate of 200 µl/min. Cells were detected by a cooled charge-coupled device camera (CH250/KAF-1400, Photometrics, Tuscon, AZ, USA) coupled to an inovision imaging system (Durham, NC, USA) and Fura-2-fluorescence was measured with an inverted fluorescence microscope (Axiovert 135 TV, Zeiss, Göttingen, Germany). Alternating excitation wavelengths, 340 nm, were delivered by a SPEX fluorolog 2 CM1T11I spectrofluorometer (SPEX Industries, Edison, NJ, USA) to excite Fura-2 AM. On the emission side, a filter was used to monitor emission wavelengths at 505 nm. Fluorescence intensity was discerned by an ISEE software for UNIX (Inovision) and data stored in the system computer.

*Electrophysiology*

Whole-cell currents and membrane potentials were recorded with a patch-clamp technique [5] by using an EPC-10 patch-clamp amplifier (HEKA Electronics, Lambercht, Germany). For details see SI. Borosilicate pipettes were polled by using a P-2000 laser puller (Sutter Instruments, CA, USA), pipettes had a resistance of 3-5 Mohm in the solutions used. Whole-cell current traces were displayed with upward deflections denoting outward currents, and the extracellular solution contained (in mmol/l): 138 NaCl, 5.6 KCl, 1.2 MgCl_2_, 2.6 CaCl_2_, and 5 HEPES-NaOH (pH 7.4). The pipette solution contained (in mmol/l); 125 KCl, 1 MgCl_2_, 10 EGTA, 25 KOH, and HEPES-KOH (pH set to 7.15). In whole-cell current recordings cells were voltage-clamped at -80 mV and subsequently depolarized in steps of 20 mV for 100 ms to +80 mV. The β-cell membrane potential was monitored using the perforated patch configuration with a pipette solution consisting of (in mmol/lM); 10 KCl, 76 K_2_SO_4_, 10 NaCl, 1 MgCl_2_, and 10 HEPES-NaOH (pH 7.35) and 200 mg of amphotericin B per ml (dissolved in Me_2_SO, final concentration of Me_2_SO less than 0.1%). Membrane potential recording was started when R_s_ < 60 Mohm. Pancreatic β-cells were identified on the basis of, firstly a cell diameter of around 15-20 μm, and secondly, a membrane potential of close to -60 mV in 3 mmol/l glucose during membrane potential recordings. All experiments were carried out at room temperature.

**References**

1. Persson AS, Klement G, Almgren M, Sahlholm K, Nilsson J, et al. (2005) A truncated Kv1.1 protein in the brain of the *megencephaly* mouse: expression and interaction. BMC Neurology 6: 65.
2. Asfari M, Janjic D, Meda P, Li G, Halban P A, et al. (1992) Establishment of 2-mercaptoethanol-dependent differentiated insulin- secreting cell lines. Endocrinology 130: 167 178.
3. Lacy PE, Kostianovsky M (1967) Method for the isolation of intact islets of Langerhans from the rat pancreas. Diabetes 16: 35 39.
4. Cavallari G, Zuellig RA, Lehmann R, Weber M, Moritz W (2007) Rat Pancreatic Islet Size Standardization by the “Hanging Drop” Technique. Transplantation. Proceedings 39: 2018 2020.
5. Hamill OP, Marty A, Neher E, Sakmann B, Sigworth FJ (1981) Improved patch-clamp techniques for high-resolution current recording from cells and cell-free membrane patches. Pflugers Arch 391: 85 10.
